# Oncogenic Osteomalacia with Elevated Fibroblast Growth Factor 23: A Rare Case of Paranasal Sinus Tumor Onset

**DOI:** 10.7759/cureus.4919

**Published:** 2019-06-17

**Authors:** Mario Rigante, Antonella Loperfido, Gaetano Paludetti

**Affiliations:** 1 Otolaryngology Institute-Department of Head and Neck, Fondazione Policlinico Universitario A. Gemelli Irccs Università Cattolica Del Sacro Cuore, Rome, ITA

**Keywords:** paranasal sinus tumor, oncogenic osteomalacia, fibroblast growth factor 23, ossifying fibromyxoid tumor, hypophosphatemia

## Abstract

Tumor-induced osteomalacia, also known as oncogenic osteomalacia, is a rare, acquired paraneoplastic disease characterized by hypophosphatemia and renal phosphate wasting.

We report on the case of a 52-year-old-man admitted to our hospital for bone and muscular pains and difficulty in walking. He underwent a computed tomography (CT) scan of the legs that documented fractures in the right tibia, femur, and fifth metatarsus. Laboratory findings showed hypophosphatemia and elevated levels of parathyroid hormone (PTH). The first diagnosis was osteomalacia, treated with calcium and vitamin D, without any benefit. So he underwent a whole body CT scan, showing a small expansive lesion occupying the left frontal sinus. Furthermore, we found high serum levels of fibroblast growth factor 23 (FGF23) using the enzyme-linked immune sorbent assay (ELISA) assay. The patient underwent endoscopic surgical resection of the frontal tumor with complete clinical remission and the histopathological diagnosis of an ossifying fibromyxoid tumor.

This is a rare case of oncogenic osteomalacia due to a paranasal sinus tumor. The main symptoms are not associated with nasal sinus involvement but with over-expressed FGF23. To conclude, physicians should never underestimate the chance of paraneoplastic syndrome in the head and neck district, even if such an occurrence is uncommon in this location. The clinical symptoms may be aspecific and not related to nose problems, making the differential diagnosis very difficult.

## Introduction

Tumor-induced osteomalacia (TIO), also known as oncogenic osteomalacia (OOM), is a rare, acquired paraneoplastic disease characterized by hypophosphatemia and renal phosphate wasting. TIO can be associated with mesenchymal tumors, which may be benign or malignant in rare cases. These mesenchymal tumors over-express fibroblast growth factor 23 (FGF23), responsible for hypophosphatemia and phosphaturia. Physiologically, FGF23 is the key regulator of phosphate metabolism [1]. Excessive FGF23 action causes several hypophosphatemic diseases, whereas deficient FGF23 activity results in hyperphosphatemic tumoral calcinosis [2]. Within the reported cases of TIO, 42.9% originate from soft tissues and 57.1% from the bone. Most tumors are reported to occur in the thigh and femur (22.7%), facies and skull (20.7%), ankle and foot (8.8%), pelvis (8.2%), tibia and fibula (6.5%), and arm (6.5%). Some tumors are even located in the liver, tongue, thyroid, and lung [3]. Head and neck regions are concerned in 5%-10 % of cases [4] and the prior sinonasal localization is extremely rare [5].

## Case presentation

**Figure 1:**
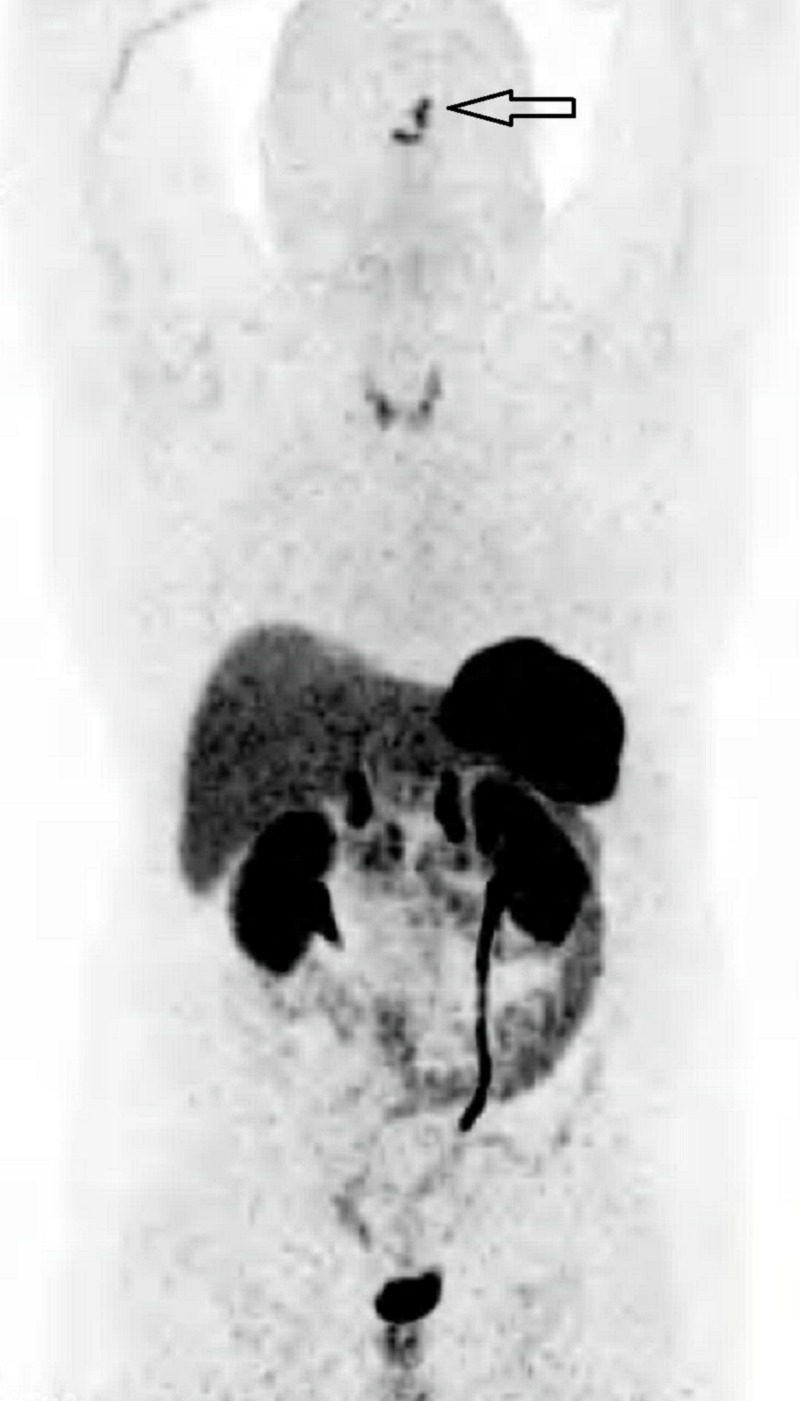
Whole Body FDG-PET/CT Evidence of a small lesion in the left frontal sinus with intense FDG uptake compatible with the recurrence of a bone lesion FDG-PET/CT: 18F-fluorodeoxyglucose positron emission tomography-computed tomography

A 52-year-old-man was admitted to our hospital for bone and muscular pains and difficulty in walking. He underwent a computed tomography (CT) scan that documented fractures in the right tibia and femur, some ribs, and the fifth metatarsus. Laboratory findings showed hypophosphatemia and elevated levels of parathyroid hormone (PTH). The first therapeutic approach was administering calcium and vitamin D without success. So he underwent a complete serum examination panel, which found high serum levels of FGF23, and a whole body CT scan showing a small expansive lesion occupying the left frontal sinus with a shell of bone detectable around the lesion. A subsequent whole-body 18F-fluorodeoxyglucose positron emission tomography-computed tomography (FDG-PET/CT) showed a small lesion in the left frontal sinus with intense FDG uptake (Figure 1).

**Figure 2:**
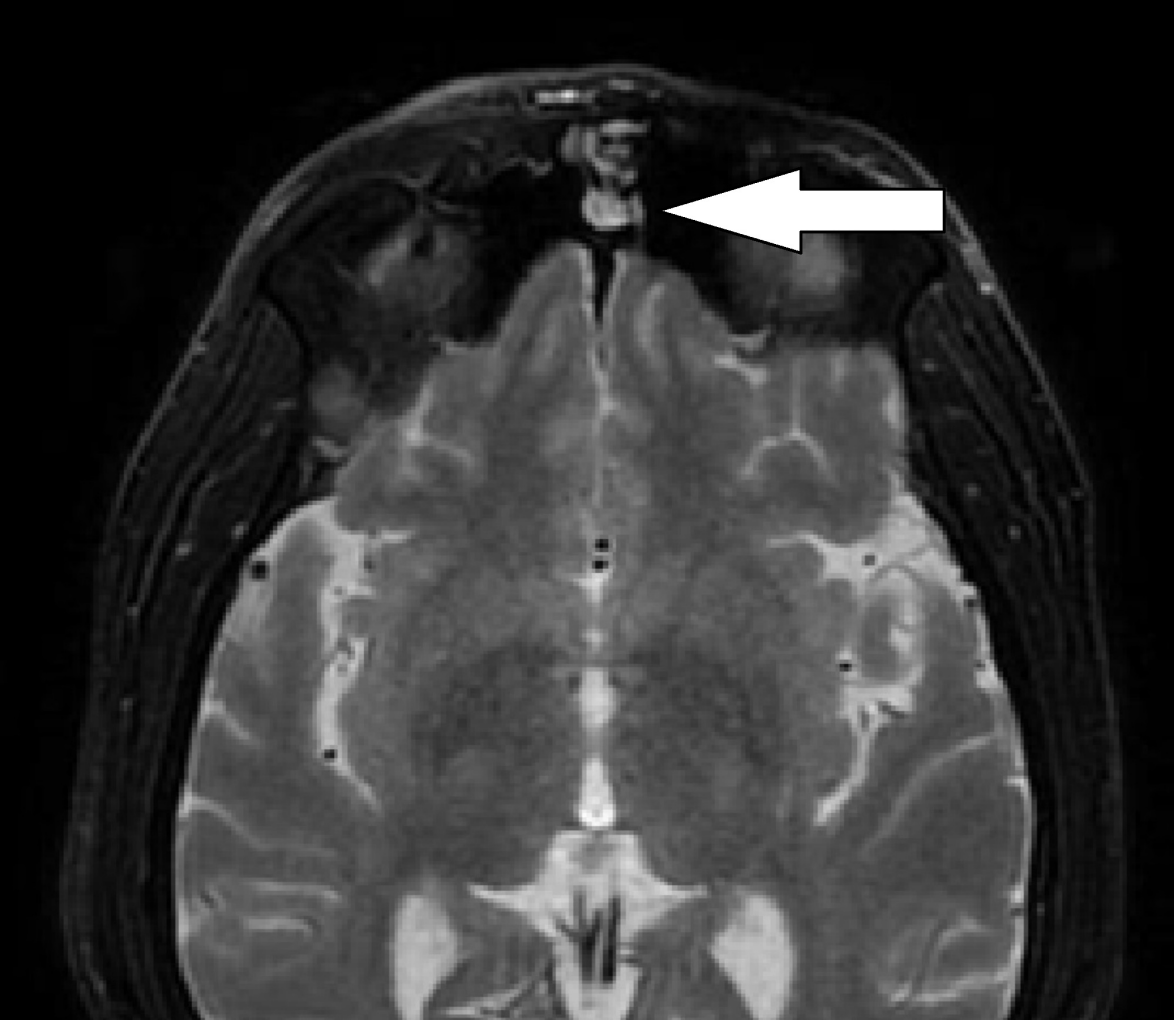
MRI T2-Weighted with Contrast Evidence of a 7 mm mass protruding in the left frontal sinus MRI: magnetic resonance imaging

Magnetic resonance imaging (MRI) showed a 7 mm mass protruding the frontal sinus (Figure 2).

**Figure 3:**
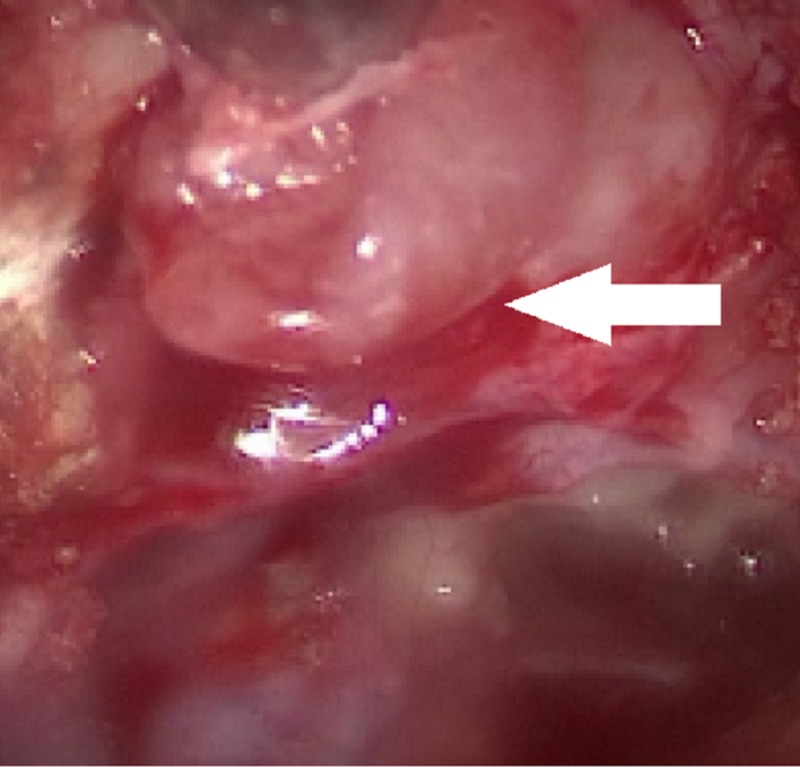
Endoscopic Frontal Sinus Surgery Endoscopic appearance of the smooth non-ulcerated lesion originating from the posterior wall of the frontal sinus

The patient underwent endoscopic removal of the lesion (Figure 3).

**Figure 4:**
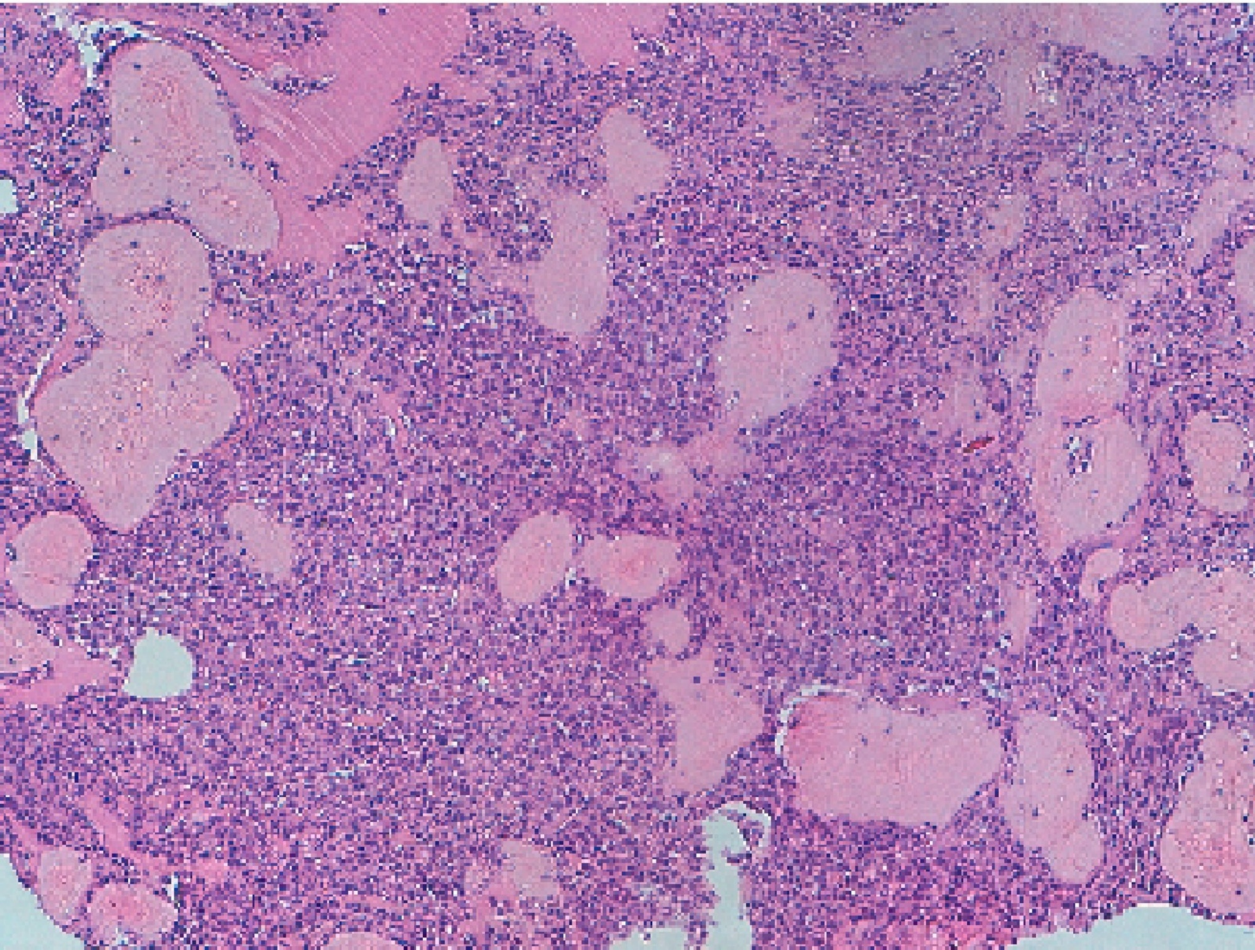
Histopathology Report Histopathologic images showing the cell neoplasm constituted by small, round, and oval elements, with mild nuclear pleomorphism and scant cytoplasm immersed in the stroma with abundant collagen, a chondroid pattern in some areas, and osteoid formation.

Histopathologic images of the excised nasal mass are shown in Figure 4.

A histological examination of the removed lesion revealed respiratory mucosa and cell neoplasm constituted by small, round, and oval elements, with nuclear pleomorphism and scant cytoplasm immersed in the stroma with abundant collagen, a chondroid pattern in some areas, and osteoid formation. There was diffuse cytoplasmic positivity for vimentin, focal and weak positivity for S100 protein, and negativity for NSE, CD10, GFAP, CD31, CD34, LCA, ASMA, SMMHC, desmin, CD99, bcl2, and AE1/AE3. Morphological and immunohistochemical features were compatible with an ossifying fibromyxoid tumor.

The patient underwent functional endoscopic sinus surgery, including frontal sinusotomy, with resection of the tumor and consequent clinical improvement, normal values of serum phosphate, and FGF23 disappearance.

Since the lesion may be classified as a mesenchymal tumor of borderline malignancy, a clinical-radiological follow-up with FDG PET/CT and laboratory findings was started. After two years, all findings were negative.

## Discussion

The differential diagnosis for an isolated sinus mass with recurrent fractures and muscular pain may include a paranasal sinus tumor with bone metastases, osteoporosis associated with a paranasal tumor, or a paranasal tumor in case of rickets and oncogenic osteomalacia. Paranasal sinus tumors rarely metastasize in bones [6-7]. Generally, this tumor occurs in males while osteoporosis is a typical postmenopausal women disease. Nutritional vitamin D deficiency is a common cause of osteomalacia in adults. Populations at risk include the homebound elderly who have little sun exposure and insufficient dietary calcium and vitamin D, patients with malabsorption related to gastrointestinal bypass surgery or celiac disease, and immigrants to cold climates from warm climates [8], but our patient was not included in such categories. Rarely, it is possible to observe paraneoplastic syndrome, such as oncogenic osteomalacia caused by a paranasal sinus bone tumor, so taking into account the abnormal value of FGF23, we ruled out the previous diagnostic hypothesis in favor of oncogenic osteomalacia.

Concerning the diagnosis, imaging modalities, such as octreotide scintigraphy, CT, MRI, and PET, are helpful to locate the tumor responsible for TIO. The specific biochemical pattern consists of hypophosphatemia, elevated levels of alkaline phosphatase, normal or low levels of 1,25-dihydroxyvitamin D, and normal circulating levels of calcium. PTH level is normal but, sometimes, it can be particularly high, reflecting secondary hyperparathyroidism, which is a normal response to low 1,25-vitamin D caused by elevated FGF23. Patients with TIO present progressive and nonspecific symptoms: bone pain, muscle weakness, reduced height, and multiple fractures, primarily in the ribs, vertebral bodies, and femoral neck [9]. The diagnosis is very difficult because the symptoms are nonspecific and they may not involve the sinonasal region: typical time from the onset of symptoms to a presumptive diagnosis of tumor-induced osteomalacia is often more than 2.5 years. Physicians should be alerted to order serum/urine phosphorus panels in patients with progressive weakness, bone and muscle pain, and pathologic fractures. In case of phosphaturia and hypophosphatemia, the potential causes of phosphate-wasting syndromes, including TIO, should be taken into account.

The treatment for TIO is surgical tumor removal, but recurrences have been reported [10]. Therefore, in case of incompletely resected tumors, adjuvant radiotherapy can be used but data are limited. Furthermore, medical treatment with phosphate supplements or active vitamin D increases the serum phosphate levels of the patient. One of the most promising treatments is blocking FGF23 by administering an anti-FGF23 antibody. These antibodies are currently being tested for other phosphaturic conditions where they safely and efficiently increased renal phosphate reabsorption and serum phosphate levels [11].

## Conclusions

In conclusion, physicians should never underestimate the chance of paraneoplastic syndrome in the head and neck district, even if such an occurrence is uncommon in this location. The clinical symptoms may be aspecific and not related to nose problems, making the differential diagnosis very difficult.
